# Bilateral Optic Disc Swelling as a Plausible Common Ocular Sign of Autoinflammatory Diseases: Report of Three Patients with Blau Syndrome or Cryopyrin-Associated Periodic Syndrome

**DOI:** 10.3390/life11121433

**Published:** 2021-12-19

**Authors:** Toshihiko Matsuo, Masato Yashiro, Osamu Yamasaki, Takehiro Tanaka, Akira Manki

**Affiliations:** 1 Graduate School of Interdisciplinary Science and Engineering in Health Systems, Okayama University, Okayama City 700-8558, Japan; 2 Department of Ophthalmology, Okayama University Hospital, Okayama City 700-8558, Japan; 3 Department of Pediatrics, Okayama University Hospital, Okayama City 700-8558, Japan; yashiro@md.okayama-u.ac.jp; 4 Melanoma Center, Department of Dermatology, Graduate School of Medicine, Dentistry, and Pharmaceutical Sciences, Okayama University, Okayama City 700-8558, Japan; yamasa-o@cc.okayama-u.ac.jp; 5 Department of Pathology, Graduate School of Medicine, Dentistry, and Pharmaceutical Sciences, Okayama University, Okayama City 700-8558, Japan; takehiro@md.okayama-u.ac.jp; 6 Department of Pediatrics, Okayama City Hospital, Okayama City 700-8557, Japan; akira_manki@okayama-gmc.or.jp

**Keywords:** autoinflammatory diseases, Blau syndrome, Muckle-Wells syndrome, CINCA/NOMID syndrome, cryopyrin-associated periodic syndromes, optic disc swelling (optic papillitis)

## Abstract

The aim of this study is to describe bilateral optic disc swelling in three consecutive patients with Blau syndrome or cryopyrin-associated periodic syndrome at a single institution. Case 1 was a 30-year-old woman receiving 25 mg etanercept twice weekly who had been diagnosed as early-onset sarcoidosis by biopsy of skin rashes at 5 months old and genetically diagnosed with Blau syndrome with *CARD15*/*NOD2* mutation (N670K) at 13 years old. At 10 years old, she began to have uveitis with optic disc swelling in both eyes, resulting in macular degeneration and optic disc atrophy at 17 years old only when etanercept was introduced. Case 2 was a 21-year-old man receiving adalimumab every 2 weeks who had been diagnosed as early-onset sarcoidosis by biopsy of skin rashes at 1.5 years old and genetically diagnosed as Blau syndrome with *CARD15*/*NOD2* mutation (C495Y) at 5 years old. At 8 years old, around the time of adalimumab introduction, he began to show bilateral optic disc swelling which continued until the age of 16 years when the dose of adalimumab was increased. Case 3 was a 20-year-old woman receiving canakinumab every 8 weeks for systemic symptoms such as fever, headache, vomiting, and abdominal pain and later for sensorineural hearing disturbance on both sides. She had been diagnosed genetically with cryopyrin-associated periodic syndrome with *NLRP3* mutation (Y859C) at 7 years old. At 5 years old, she was found to have bilateral optic disc swelling, which continued until the age of 10 years when she began receiving canakinumab (IL-1β inhibitor). Bilateral optic disc swelling might be tentatively designated as a plausible common ocular feature, if it occurred, in autoinflammatory diseases to pay more attention to ophthalmic complications in rare diseases.

## 1. Introduction

Autoinflammatory diseases are a recently established collection of monogenic diseases, which are characterized by abnormal activation of the innate immune system [1,2,3]. In the entity of autoinflammatory diseases, familial Mediterranean fever is most common in the sub-entity of periodic fever diseases, which show remittent fever, skin rashes, and joint pain. Cryopyrin-associated periodic syndromes, which have been called cryopyrinopathies, follow next at the rate of diseases in the sub-entity of periodic fever diseases. The spectrum of cryopyrin-associated periodic syndromes consists of clinical diagnoses of familial cold autoinflammatory disease, Muckle–Wells syndrome, and chronic infantile neurological cutaneous and articular (CINCA)/neonatal onset multisystem inflammatory disease (NOMID) syndrome. The other sub-entities in autoinflammatory diseases are pyogenic disorders and granulomatous disorders, including Blau syndrome.

From the viewpoint of molecular mechanism, defects of interleukin-1β (IL-1β) family regulation underlay the pathogenesis of familial Mediterranean fever and cryopyrin-associated periodic syndromes. More specifically, cryopyrin-associated periodic syndromes are caused by gain-of-function mutations in the *NLRP3* gene, which lead to overproduction of a proinflammatory cytokine, IL-1β. Blau syndrome, previously called early-onset sarcoidosis, is caused by gain-of-function mutations in the *NOD2* gene and characterized as a disease linked to nuclear factor-κB (NF-κB) activation, leading to overexpression of inflammatory genes, such as tumor necrosis factor-α (TNF-α). Based on the molecular pathophysiology, molecular target drugs have now been used to control autoinflammatory diseases [1,2,3].

Most frequent eye manifestations in autoinflammatory diseases may be conjunctivitis [4,5]. In this study, we focused on bilateral optic disc swelling, which happened to be shared by three consecutive Japanese patients with Blau syndrome or cryopyrin-associated periodic syndrome at our single institution. The optic disc swelling in both eyes might be a plausible common eye manifestation, if it occurred as a complication, in autoinflammatory diseases. In this study, we report the ocular and systemic manifestations in these three patients to enhance the understanding of eye complications of the rare diseases among pediatricians and ophthalmologists as a subject for further evaluation.

## 2. Case Reports

### 2.1. Case 1

**Figure 1:**
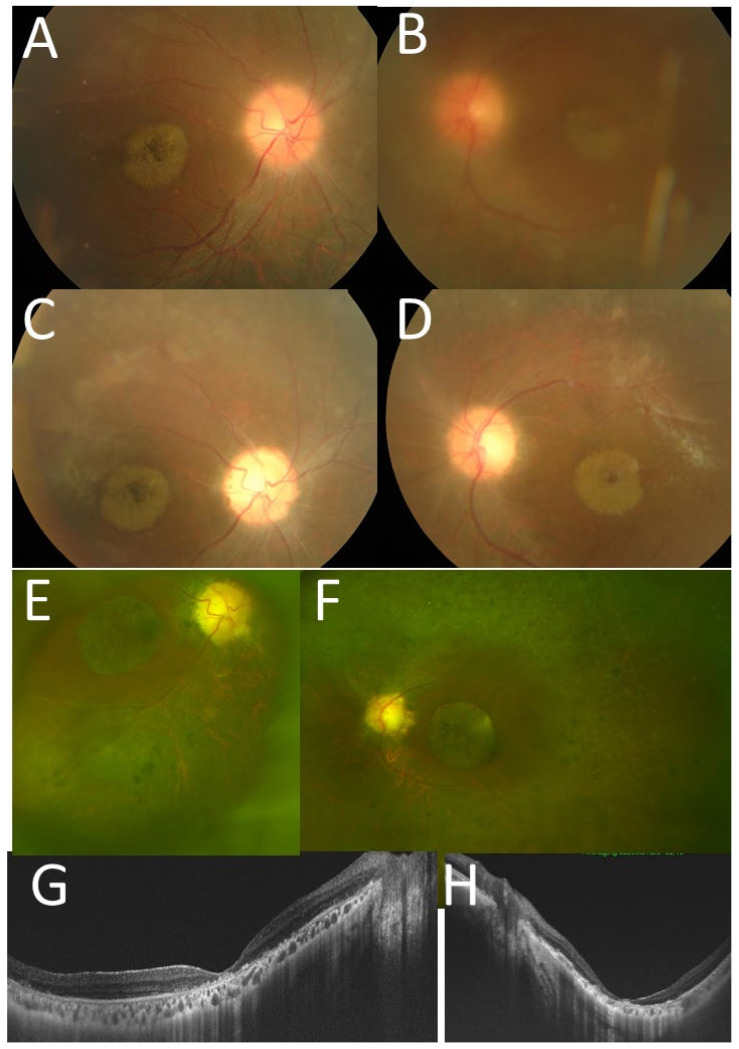
Case 1. Fundus photographs at the age of 17 years ((**A**), right eye; (**B**), left eye), at the age of 18 years ((**C**), right eye; (**D**), left eye), and at the age of 30 years ((**E**), right eye; (**F**), left eye). The optic discs in both eyes are hyperemic and blurred at 17 years (**A**,**B**), but become pale tone and atrophic at 30 years with macular degeneration as well as diffuse midperipheral retinal degeneration. Optical coherence tomography at 30 years ((**G**), right eye; (**H**) left eye) shows the thinned retina at the macula in both eyes.

A 30-year-old woman with Blau syndrome was systemically stable with oral thalidomide 150 mg/day, prednisolone 8 mg/day, tacrolimus hydrate 1 mg/day, loxoprofen 60 mg/day, methotrexate 8 mg/week, and subcutaneous injection of 25 mg etanercept twice weekly. Hypertension was controlled with benidipine (4 mg/day) and imidapril (5 mg/day). She was short and obese with a height of 131 cm and body weight of 101 kg. Genetic testing at the age of 13 years revealed a heterozygous missense mutation in the NOD-2 region of the *CARD15*/*NOD2* gene (N670K) [6,7,8,9,10]. Ophthalmologically, the best-corrected visual acuity was hand movement in the right eye and 0.01 in the left eye. The intraocular pressure in both eyes was 10 mmHg. She had intraocular lens implantation in both eyes with no aqueous cells and no keratic precipitates. She had optic disc atrophy, macular degeneration, and diffuse retinal degeneration of midperipheral fundus in both eyes as a sequel to uveoretinitis (Figure 1E–H). She had no intraocular inflammation with 0.1% betamethasone, four times daily, and 0.1% bromfenac, twice daily.

**Figure 2:**
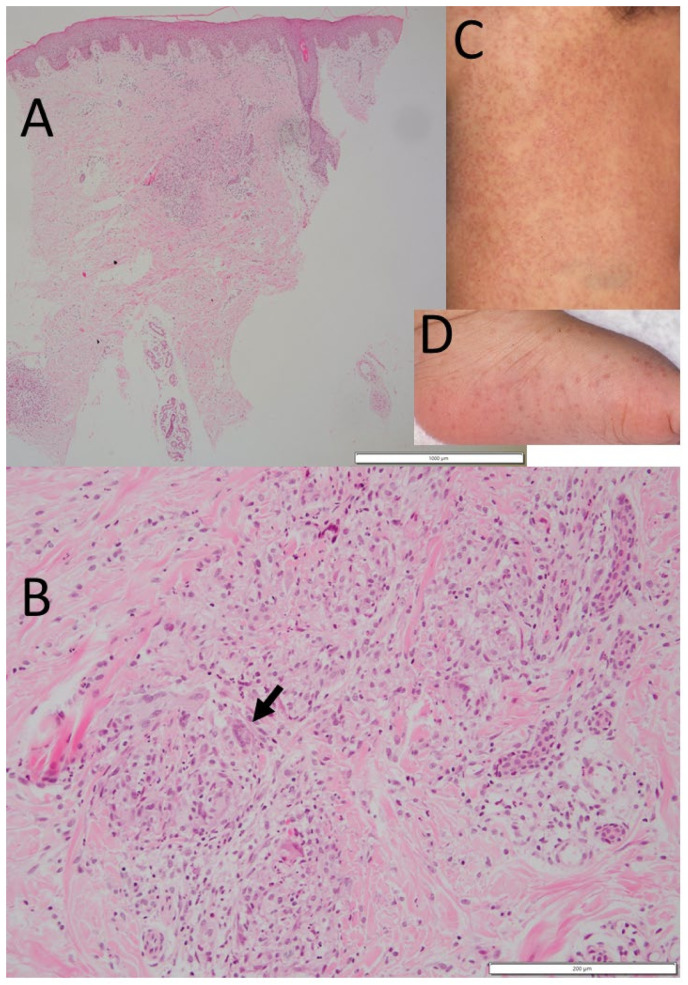
Case 1. Skin rashes (**C**,**D**) and biopsy (**A**,**B**) at the age of 5 months. Subcutaneous non-caseating granuloma with multinucleated giant cells (arrow in (**B**)) and epithelioid cells. Red papules on the back (**C**) and left foot sole (**D**). Scale bar = 1000 μm in (**A**), 200 μm in (**B**).

In her past history, at the age of 5 months, she began to have papules on the trunk (Figure 2C), and then in one month, papules also developed in the upper and lower extremities on both sides (Figure 2D). She had fever up to 39.8 °C but had no arthropathy at that time. Biopsy of the papules revealed non-caseating granuloma with multinucleated giant cells (Figure 2A,B) [6]. Blood angiotensin-converting enzyme was elevated to 25.5 IU/L and lysozyme elevated to 14.3 μg/mL. In 3 months, the papules resolved spontaneously to leave mild pigmentation.

At 2 years old, she had low-grade fever up to 37.7 °C, bilateral cervical, submandibular, and inguinal small lymphadenopathy, sausage-like swelling of bilateral fingers as well as painful arthropathy involving bilateral wrists, knees, ankles, and toes. Plain X-ray films revealed no bony destruction in the joints. White blood cell count was 13 × 10^3^/μL, C-reactive protein 5.8 mg/dL, and the erythrocyte sedimentation rate was 48 mm/1 h. Eye examinations revealed no uveitis. Right inguinal lymph node biopsy showed reactive lymphoid hyperplasia. An oral prednisolone dosage of 2 mg/kg body weight resolved lymphadenopathy and painful arthropathy within a few days. Oral prednisolone was tapered and discontinued in 2 months. However, oral prednisolone was resumed at the age of 3 years. At this time, she received oral prednisolone at 25 mg every other day, and began to show small retinal flecks and snowball-like vitreous opacity in the inferior fundus of both eyes. In the following 4 years, she showed stable retinal manifestations with retinal periphlebitis and retinal spotty exudates in the inferior fundus of both eyes and maintained the visual acuity of 1.2 in both eyes. No corticosteroid eye drops were used since she had no anterior chamber inflammation. At the age of 9.5 years, the administration of oral prednisolone was changed to a daily dose of 17 mg. At 10.5 years old, cyclosporine 40 mg/day was combined with oral prednisolone 10 mg/day.

**Figure 3:**
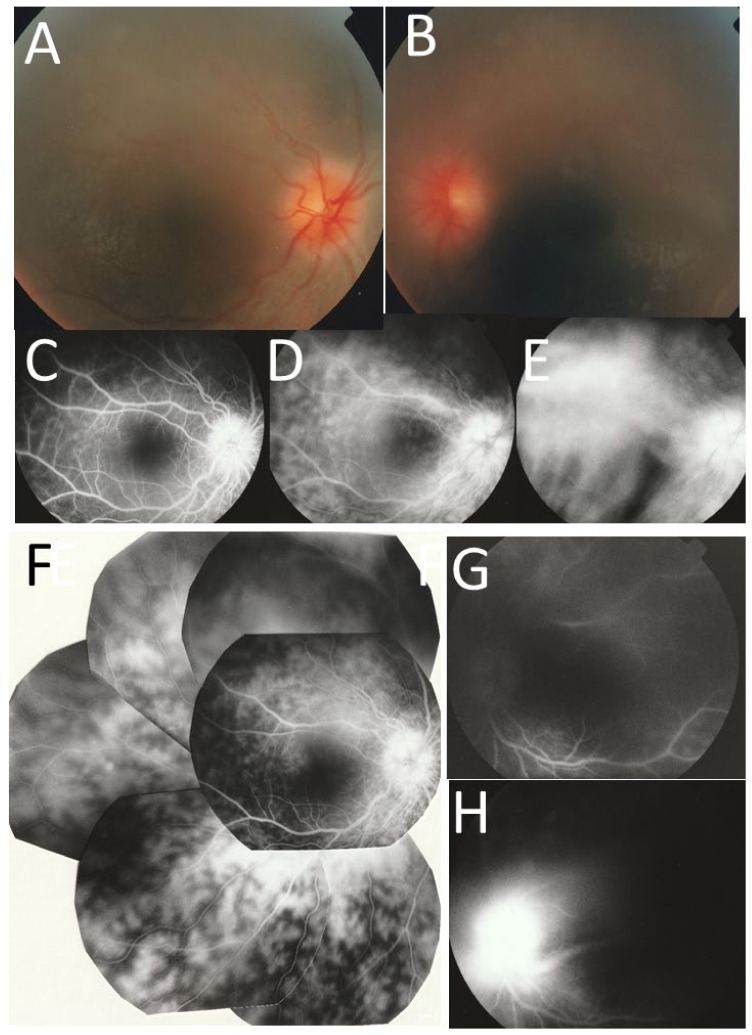
Case 1. Fundus photographs ((**A**), right eye; (**B**), left eye) showing hyperemic swollen optic discs in both eyes at the age of 10 years. Fluorescein angiography in the right eye ((**C**) at 22 s, (**D**) at 1 min 53 s, (**E**) at 5 min 24 s after fluorescein dye injection at cubital vein, (**F**), merged) and in the left eye ((**G**) at 1 min 5 s, (**H**) at 5 min 49 s).

At the age of 10 years and 9 months when the dose of oral prednisolone was at 6 mg daily in combination with cyclosporine 120 mg/day, she showed 2+ aqueous cells and 1+ mutton-fat keratic precipitates in both eyes. The best-corrected visual acuity was 0.6 in the right eye and 1.0 in the left eye. The intraocular pressure was 19 mmHg in both eyes. She had steroid-induced posterior subcapsular cataract in both eyes and optic disc swelling with diffusely edematous retina in the midperipheral fundi of both eyes (Figure 3A,B). Fluorescein angiography showed optic disc capillary dilation and leakage with diffuse retinal capillary leakage in the midperipheral fundi of both eyes (Figure 3C–H). Topical 0.1% betamethasone eye drops 4 times daily was started, and oral prednisolone 6 mg daily was increased to 30 mg daily in consultation with a pediatrician. In the following 3 months, the optic disc swelling and aqueous cells subsided and oral prednisolone was tapered to 15 mg daily. The best-corrected visual acuity was 1.0 in the right eye and 1.5 in the left eye.

The best-corrected visual acuity in the right eye showed a decrease from 0.7 to 0.1 in 2 months around 12 years old, and finally to 0.02 in the following 2 months, due mainly to the deterioration of cataract. At the age of 12 years and 8 months when oral prednisolone was 7 mg daily, she underwent cataract surgery with intraocular lens implantation in the right eye, leading to the visual acuity increase from 0.02 to 0.2 in the right eye. At 13 years old, oral prednisolone was increased to 40 mg daily due to 3+ aqueous cells and optic disc swelling in both eyes. The intraocular inflammation subsided, and oral prednisolone was tapered to 8 mg daily in one year at 14 years old. The visual acuity was 0.1 in the right eye and 0.7 in the left eye. At 15 years old, oral cyclosporine was replaced with oral tacrolimus 2 mg/day, which was increased to 3 mg/day in 3 months.

At 16 years old, she began to take oral thalidomide 100 mg/day since she had severe systemic arthralgia and persistent uveitis with optic disc swelling in both eyes (Figure 1A,B). In a month, arthralgia subsided and C-reactive protein returned to the normal level. The best-corrected visual acuity was 0.3 in the right eye and 0.2 in the left eye, but decreased to 0.1 in the right eye and 0.01 in the left eye in several months. She underwent cataract surgery with intraocular lens implantation in the left eye. The best-corrected visual acuity after the surgery was 0.1 in both eyes. At the age of 17 years and 10 months, she began to have subcutaneous injection of etanercept 25 mg twice in a week, and one year later, oral methotrexate 8 mg/week was combined to have stable disease thereafter (Figure 1C,D). The visual acuity, however, gradually decreased to 0.01 in both eyes due to macular degeneration and optic disc atrophy in 10 years.

### 2.2. Case 2

**Figure 4:**
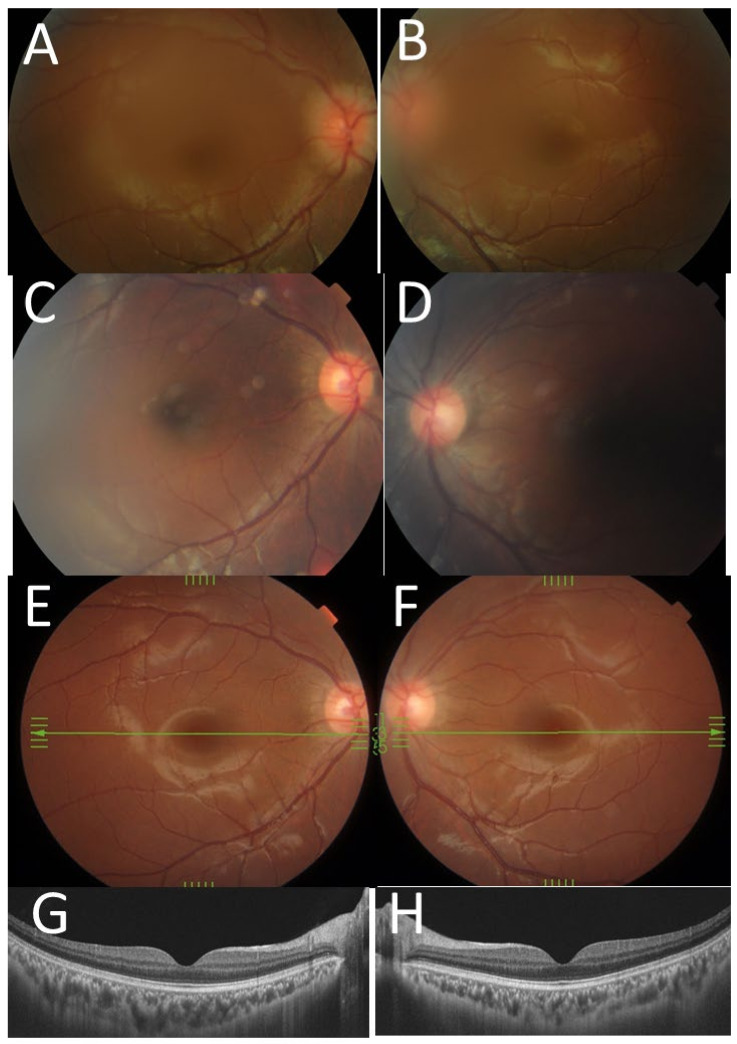
Case 2. Fundus photographs ((**A**), right eye; ((**B**), left eye) at the age of 8 years showing hyperemic swollen optic discs in both eyes. The optic discs in both eyes appear normal at the age of 16 years ((**C**), right eye; (**D**), left eye) and at the age of 19 years ((**E**), right eye; (**F**), left eye). Optical coherence tomography shows the normal macula in both eyes ((**G**), right eye; (**H**), left eye) at the age of 20 years.

A 21-year-old man with Blau syndrome was systemically stable receiving 200 mg/day of oral thalidomide, prednisolone (5 mg/day), tacrolimus hydrate (1 mg/day), methotrexate (10 mg/week), and subcutaneous injection of 40 mg adalimumab every 2 weeks. He had a short stature with a height of 129 cm but no obesity with a weight of 35 kg. Genetic testing at the age of 5 years revealed a heterozygous missense mutation in the NOD-2 region of the *CARD15*/*NOD2* gene (C495Y) [7,8,9,10]. Ophthalmologically, the best-corrected visual acuity was 1.2 in the right eye and 0.9 in the left eye. The intraocular pressure was 9 mmHg in both eyes. He had intraocular lens implantation in both eyes and showed neither aqueous cells nor keratic precipitates in both eyes. The optic discs in both eyes were normal and the retina had no abnormalities (Figure 4E–H). Mild posterior capsular opacity (aftercataract) could explain slightly reduced visual acuity in the left eye. He used no eye drops because of no intraocular inflammation in both eyes.

**Figure 5:**
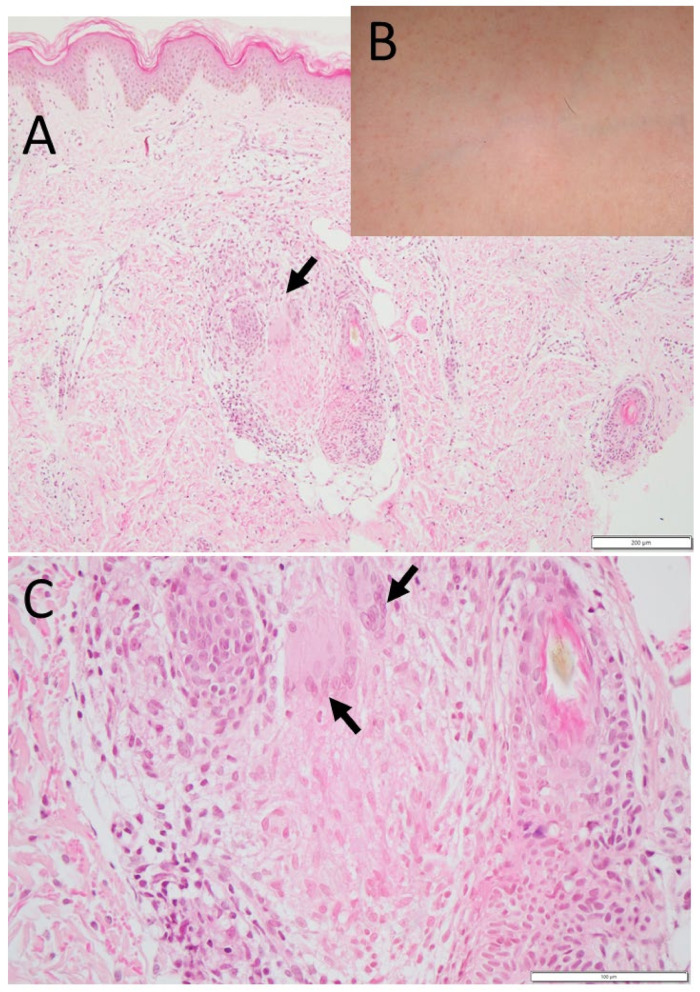
Case 2. Skin rashes (**B**) and biopsy (**A**,**C**) at the age of 1.5 years. Subcutaneous non-caseating granuloma with multinucleated giant cells (arrows in (**A**,**C**)) and epithelioid cells. Red papules on the chest (**B**). Scale bar = 200 μm in (**A**), 100 μm in (**C**).

In his past history, at the age of 1.5 years, he developed papules on the trunk (Figure 5B) and upper limbs, with low-grade fever at 37 °C and bilateral finger swelling. Skin biopsy revealed non-caseating granuloma with multinucleated giant cells (Figure 5A,C). Eye examinations showed no intraocular inflammation. Arthropathy resolved and C-reactive protein became undetectable with dosages of oral prednisolone of 9 mg/day and cyclosporine of 100 mg/day. At the age of 3 years and 4 months, he began to take 6 mg/week of oral methotrexate. At 4 years old, he developed bilateral lower lid tumors suspected of granuloma, which resolved spontaneously in 4 months.

At 8 years old, he began to have subcutaneous injection of 20 mg adalimumab every 2 weeks. At the age of 8.5 years, he continued to show skin eruption, remittent fever, and arthropathy of knee, hip, wrist, and finger joints on both sides. At this time for the first time, he developed optic disc swelling in both eyes (Figure 4A,B), but he did not show afferent pupillary detect or visual acuity decrease in either eye. He began to take oral thalidomide at 75 mg/day, in addition to prednisolone, cyclosporine, and subcutaneous injection of adalimumab. At 16.5 years old, the dose of adalimumab was increased to 40 mg every 2 weeks, and around this time, the optic disc swelling in both eyes almost subsided (Figure 4C,D). At 19.5 years old, he underwent cataract surgery with intraocular lens implantation in both eyes for steroid-induced cataract.

### 2.3. Case 3

**Figure 6:**
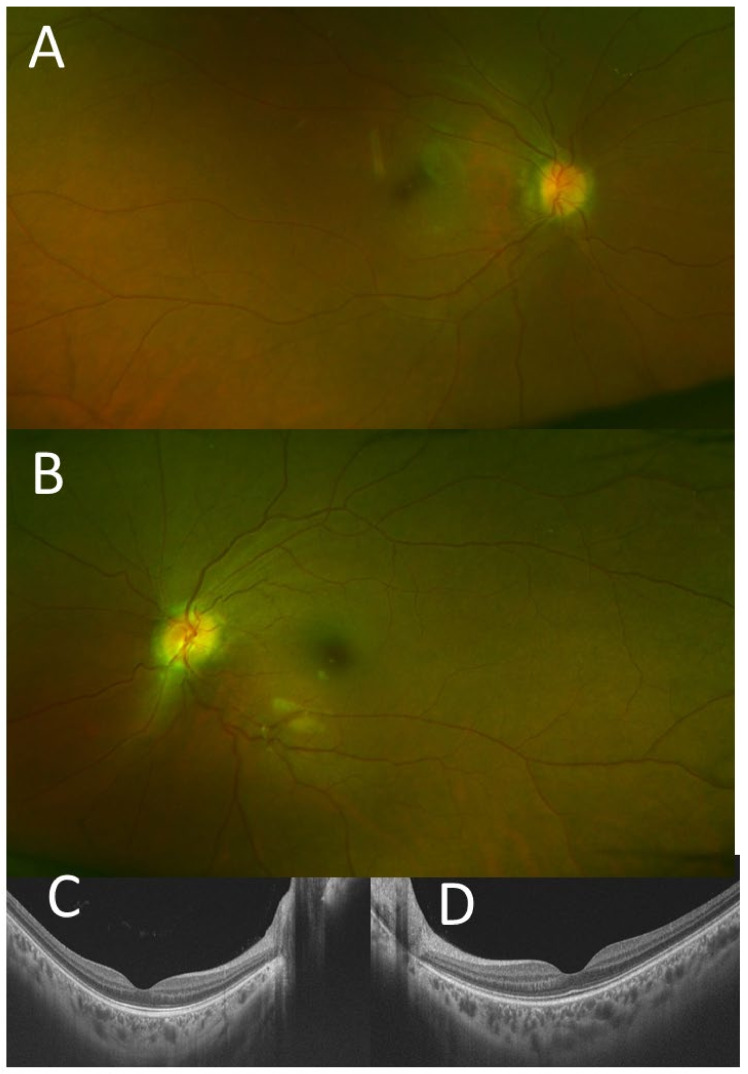
Case 3. Fundus photographs ((**A**), right eye; (**B**), left eye) showing normal optic discs and optical coherence tomography showing the normal retinal structure of the macula ((**C**), right eye; (**D**), left eye) at the age of 20 years.

A 20-year-old woman with cryopyrin-associated periodic syndrome was stable with a subcutaneous injection of 6 mg/kg body weight of canakinumab every 8 weeks to control systemic symptoms such as fever, headache, vomiting, and abdominal pain and also to slow down the deterioration of sensorineural hearing disturbance on both sides. Genetic testing at the age of 7 years revealed a heterozygous missense mutation in the exon 3 of *NLRP3* *(CIAS1)* gene (Y859C) [11]. The best-corrected visual acuity was 1.5 in both eyes, and the intraocular pressure was 16 mmHg in both eyes. She had no aqueous inflammation. The optic discs in both eyes were normal (Figure 6A–D). She had normal height at 157 cm and weight at 50 kg.

**Figure 7:**
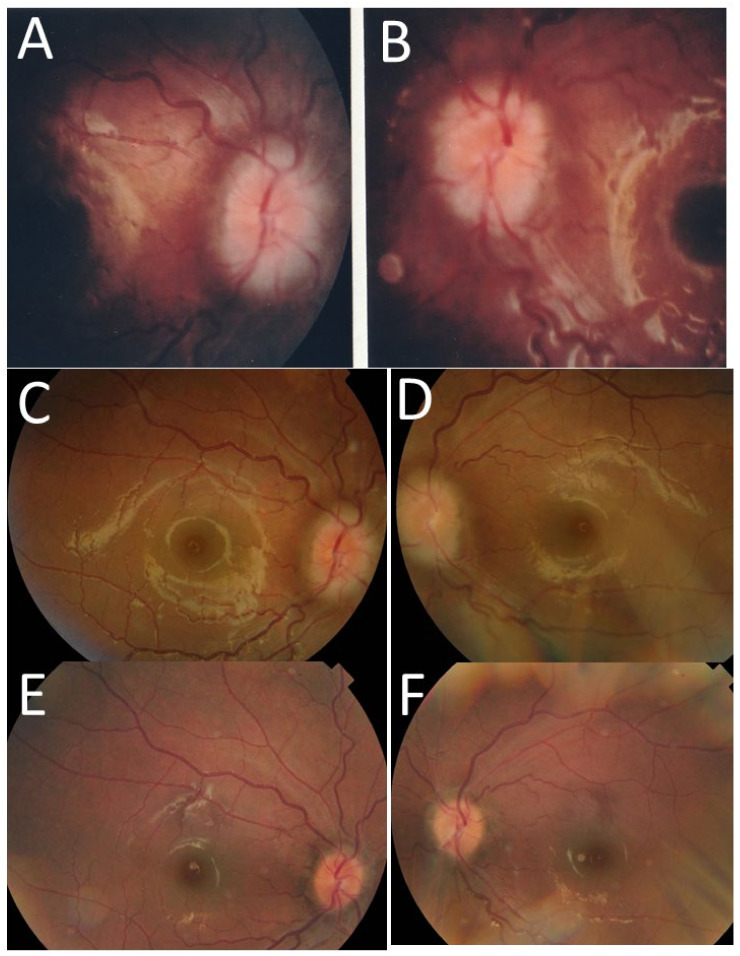
Case 3. Fundus photographs at the age of 5 years ((**A**), right eye; (**B**), left eye), 9 years ((**C**), right eye; (**D**), left eye), and 12 years ((**E**), right eye; (**F**), left eye). Blurred swollen optic discs in both eyes at the age of 5 years and 9 years have become almost normal at the age of 12 years.

In her past history, at the age of 4 years, she began to experience vomiting once a month, and persistent low-grade fever. She had no myalgia, arthralgia, or skin rashes. Eye examinations revealed no optic disc edema in either eye. Serum C-reactive protein was elevated to 2–3 mg/dL. She had exudative otitis media on both sides and was treated successfully by tympanostomy tubes. At 5 years old, she was found to have optic disc swelling in both eyes (Figure 7A,B) at the consultation of bilateral conjunctival injection. The best-corrected visual acuity was 1.0 in both eyes, and she had no inflammation in anterior chamber of both eyes. The afferent pupillary detect was not noted in either eye. Head magnetic resonance imaging disclosed no intracranial abnormality.

**Figure 8:**
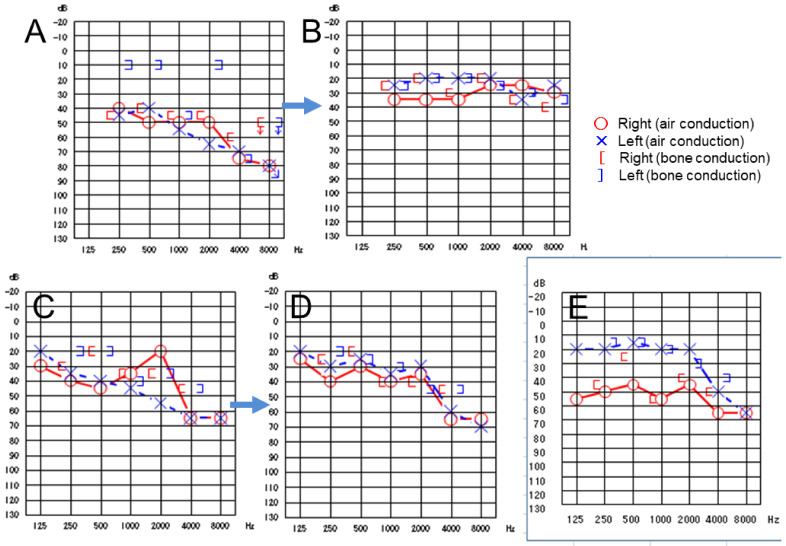
Case 3. Audiograms at the age of 7 years before ((**A**)) and after (**B**) two courses of steroid pulse therapy (arrow). Audiograms at the age of 9 years before (**C**) and after (**D**) four courses of steroid pulse therapy (arrow). Audiogram at the age of 10 years (**E**) when canakinumab was introduced.

At 6 years old, she took oral colchicine for fever, for which there was no effect after one month. Spinal tap showed protein at 29 mg/dL, glucose at 35 mg/dL, and 44 cells/μL of cerebrospinal fluid with 43% of polymorphonuclear cells, indicative of mild aseptic meningitis. At 7 years old, she underwent two courses of steroid pulse therapy with methylprednisolone for the deterioration of sensorineural hearing disturbance on both sides (Figure 8A,B). At 9 years old, she underwent 4 courses of steroid pulse therapy with methylprednisolone, but with a limited effect on hearing disturbance in high-pitch sounds on both sides (Figure 8C,D). She maintained the visual acuity of 1.2 in both eyes and the normal visual field in both eyes by Goldmann perimetric testing, but with persistent bilateral optic disc swelling (Figure 7C,D).

At the age of 9.5 years, she tried subcutaneous injection of anakinra (IL-1 receptor antagonist) every other day for a few months. Around the age of 10 years, she switched to have subcutaneous injection of canakinumab (IL-1β inhibitor) at the initial dose of 2 mg/kg body weight and then at 4 mg/kg body weight (150 mg) every 8 weeks, leading to complete resolution of bilateral optic disc swelling (Figure 7E,F). The dose of canakinumab was increased to 6 mg/kg (300 mg) at the age of 15.5 years, leading to better preservation of hearing thereafter (Figure 8E).

## 3. Discussion

A clinical question in this study was that bilateral optic disc swelling might be a common eye manifestation, if it occurred, in autoinflammatory diseases. To answer this question, we reviewed eye manifestations in three consecutive patients with Blau syndrome or cryopyrin-associated periodic syndrome at our single institution. Case 1 with Blau syndrome at the age of 6 months had been reported by dermatologists to have granulomatous skin lesions by biopsy, leading to the diagnosis of early-onset sarcoidosis [6]. Case 1 and Case 2 with Blau syndrome had been reported by pediatricians to take oral thalidomide to control systemic symptoms at the age of 16 years and 8 years, respectively [7]. The present study focused especially on eye manifestations in Case 1 and Case 2, and described their clinical features in response to etanercept and adalimumab introduction, respectively, as current standard therapy, in the disease course after the time points of the preceding report [7]. The *CARD15*/*NOD2* mutations in Case 1 and Case 2 with minimal clinical data have been reported in multi-center genetic studies of the series of Japanese patients with Blau syndrome [8,9,10]. Thus, in this study, the detailed systemic features and treatment were shown in parallel with the eye manifestations to understand the relation of ocular signs with systemic conditions.

The *NLRP3* mutation in Case 3 with no clinical data had been included in a multi-center genetic study involving Japanese patients with Muckle–Wells syndrome [11]. No detailed description of clinical features in Case 3 were reported so far. From the standpoint of clinical symptoms, Case 3 lacked urticaria-like skin rashes and arthralgia, and showed fever, headache, conjunctivitis-like injection, and sensorineural hearing disturbance. The clinical diagnosis of Case 3 was located in the spectrum of cryopyrin-associated periodic syndromes between Muckle–Wells syndrome and CINCA/NOMID syndrome [12] and appeared to be more like Muckle–Wells syndrome. The sensorineural hearing disturbance in Case 3 was key for the diagnosis of cryopyrin-associated periodic syndromes (Figure 8).

The three presented patients, including two with Blau syndrome and one with cryopyrin-associated periodic syndrome, showed a common feature of bilateral optic disc swelling as an eye manifestation. The two patients with Blau syndrome showed skin rashes, remittent fever, and arthralgia and were initially diagnosed as early-onset sarcoidosis, based on the non-caseating granulomatous lesions in skin biopsy. Of these two patients with Blau syndrome, one (Case 1) showed diffuse retinal capillaritis with optic disc swelling in both eyes at the age of 10 years (Figure 3A–H), while the other (Case 2) showed only the optic disc swelling in both eyes at the age of 8 years (Figure 4A,B). The first patient (Case 1) with diffuse retinal capillaritis resulted in diffuse retinal degeneration with macular degeneration, leading to poor vision in both eyes. The second patient (Case 2) only with bilateral optic disc swelling maintained good visual acuity throughout the course after cataract surgery.

The difference in clinical courses between the two patients (Case 1 and Case 2) with Blau syndrome would be attributed to the timing of availability of etanercept and adalimumab, TNF-α blockers, in the Japanese market. In Case 1, with poor visual outcome, etanercept was introduced at the age of 17 years when she already had macular degeneration and optic disc atrophy in both eyes. In contrast, adalimumab was introduced in Case 2 at the age of 8 years when he developed bilateral optic disc swelling. In Case 3, with cryopyrin-associated periodic syndrome, canakinumab, IL-1β inhibitor, was introduced for systemic symptoms and sensorineural hearing disturbance at the age of 10 years, and the optic disc swelling in both eyes also subsided. Regarding these three patients, afferent pupillary defect was not noted in the background of good visual acuity. In Case 1, in the later phase with poor visual acuity, the atrophic and rigid iris after uveitis resulted in the rigid pupils in both eyes.

Optic disc swelling is a manifestation of optic papillitis or choked disc caused by intracranial hypertension. After intracranial lesions, such as tumors, have been excluded by head imaging, the optic disc swelling has to be differentially diagnosed in the list of uveitis in children: juvenile idiopathic arthritis (JIA) [13,14], tubulointerstitial nephritis and uveitis (TINU) syndrome [15], and bilateral iridocyclitis with retinal capillaritis (BIRC) in juvenile [16]. In this way, autoinflammatory diseases such as Blau syndrome and periodic fever diseases must be considered in the list of differential diagnoses for childhood uveitis.

In the literature search, bilateral optic disc swelling was indeed described in Blau syndrome [17,18,19,20,21,22,23,24,25,26,27], cryopyrin-associated periodic syndromes, including Muckle–Wells syndrome and CINCA/NOMID syndrome [28,29,30,31,32,33,34,35,36], and familial Mediterranean fever [37,38]. The optic disc swelling in both eyes in these previous reports appeared to be optic papillitis and would not be attributed to intracranial hypertension. Under the circumstances, it remains rather strange that most patients, including the present two patients (Case 2 and Case 3), maintained good vision even with the optic disc swelling. Optic papillitis is usually associated with decreased visual acuity and afferent pupillary defect. This discrepancy might be the reason why most case reports used the term ‘optic disc swelling” instead of “optic papillitis”. In the present report, Case 2 and Case 3 as well as Case 1 in the earlier phase of the disease showed good visual acuity with no afferent pupillary detect in both eyes, and thus, “bilateral optic disc swelling” would be an appropriate term to describe the eye manifestation in these patients.

It remains to be determined whether bilateral optic disc swelling in autoinflammatory diseases would show the response to molecular target drugs as the current standard therapy. In the present series of patients, etanercept had an effect on the control of panuveitis in both eyes, at least in Case 1 with Blau syndrome. In contrast, the bilateral optic disc swelling as an isolated ocular manifestation in Case 2 and Case 3 might be self-limiting since the visual acuity did not decrease and the afferent pupillary defect was not noted. The maintenance of good vision in Case 2 and Case 3 might be otherwise ascribed to the early introduction of molecular target drugs, adalimumab in Case 2, and canakinumab in Case 3, in the earlier course of the diseases. Indeed, the optic disc swelling in Case 2 with Blau syndrome was associated with elevated C-reactive protein in blood as a systemic inflammatory sign while the optic disc swelling in Case 3 with cryopyrin-associated periodic syndrome would be concurrently related with aseptic meningitis.

In general, eye manifestations in autoinflammatory diseases are described roughly as conjunctivitis, anterior uveitis, and posterior uveitis in the previous reports on the series of Japanese patients with Blau syndrome [8,9,10] and Muckle–Wells syndrome [11] which also included the present three patients. Optic disc swelling would be the part of manifestations in posterior uveitis. Since optic disc swelling might be a vision-threatening condition, it is important for pediatricians and ophthalmologists to recognize the manifestation as early as possible in the disease course and to start molecular target drugs as the current standard therapy. In this context, it would be helpful for clinicians with different specialties to further evaluate that the bilateral optic disc swelling might be a plausible common eye manifestation, if it occurred as a complication of rare diseases, which are together listed in the entity of autoinflammatory diseases.

## Data Availability

Not applicable.
